# A Review of SARS-CoV2: Compared With SARS-CoV and MERS-CoV

**DOI:** 10.3389/fmed.2021.628370

**Published:** 2021-12-07

**Authors:** Huan Zhou, Junfa Yang, Chang Zhou, Bangjie Chen, Hui Fang, Shuo Chen, Xianzheng Zhang, Linding Wang, Lingling Zhang

**Affiliations:** ^1^National Drug Clinical Trial Center, The First Affiliated Hospital of Bengbu Medical College, Bengbu, China; ^2^School of Pharmacy, Bengbu Medical College, Bengbu, China; ^3^School of Public Foundation, Bengbu Medical University, Bengbu, China; ^4^Key Laboratory of Anti-inflammatory and Immune Medicine, Ministry of Education, Institute of Clinical Pharmacology, Anhui Medical University, Hefei, China; ^5^Basic Medical Sciences, Anhui Medical University, Hefei, China; ^6^Department of Oncology, The First Affiliated Hospital of Anhui Medical University, Hefei, China; ^7^Department of Pharmacology, The Affiliated Hospital of Hangzhou Normal University, Hangzhou, China

**Keywords:** COVID-19, SARS-CoV, WHO, MERS-CoV, PHEIC 3

## Abstract

The outbreak of coronavirus disease 2019 (COVID-19) has been spreading rapidly in China and the Chinese government took a series of policies to control the epidemic. Studies found that severe COVID-19 is characterized by pneumonia, lymphopenia, exhausted lymphocytes and a cytokine storm. Studies have showen that SARS-CoV2 has significant genomic similarity to the severe acute respiratory syndrome (SARS-CoV), which was a pandemic in 2002. More importantly, some diligent measures were used to limit its spread according to the evidence of hospital spread. Therefore, the Public Health Emergency of International Concern (PHEIC) has been established by the World Health Organization (WHO) with strategic objectives for public health to curtail its impact on global health and economy. The purpose of this paper is to review the transmission patterns of the three pneumonia: SARS-CoV2, SARS-CoV, and MERS-CoV. We compare the new characteristics of COVID-19 with those of SARS-CoV and MERS-CoV.

## Introduction

Coronaviruses (CoVs) are enveloped RNA viruses that respond primarily to respiratory and intestinal infections in animals and humans (1, 2). Historically, CoV infection in humans was closely linked to the human CoVs (HCoVs) HCoV-229E and HCoV-OC43-induced mild upper respiratory tract diseases (2). Nevertheless, a new fatal CoV was discovered in 2003, leading to SARS-CoV, and thus redefining historical cognition (3). More importantly, researchers have identified that respiratory diseases were significantly associated with HcoVs. For example, studies demonstrated that HCoV-HKU1 induced chronic pulmonary disease (4). HCoV-NL63 could mediate upper and lower respiratory tract diseases in children and adults around the world (5), and the recently (April 2012) Middle East Respiratory Syndrome (MERS-CoV) has been shown to be involved in acute pneumonia and sporadic renal failure (6). These findings proved that CoVs were important human pathogenic factors, and emphasize the necessity of reverse genetic systems to facilitate the genetic manipulation of the viral genome, thereby studying basic viral processes, developing candidate vaccines, and testing antiviral drugs.

In the past two decades, the world experienced outbreaks of coronavirus infection that threaten the global pandemic in 2002–2003 by SARS-CoV and in 2011 by MERS-CoV. In both cases, the causative agents (SARS-CoV and MERS-CoV, respectively), were newly identified coronavirus in the genus Beta-coronavirus with zoonotic origin the SARS-CoV (2002) and the MERS (2012) (7, 8). Moreover, studies demonstrated that SARS-CoV and MERS-CoV could infect humans, camesl, bats, cows, and civets, and thus infect the acute and fatal respiratory of humans (7, 9).

The recent coronavirus outbreak happened in the Wuhan city of China, which is known as the 2019-nCoV outbreak, recently renamed as SARS-CoV-2 outbreak or COVID-19 (10). The SARS-CoV-2 spread throughout the world is a zoonotic virus and is the seventh coronavirus that infects the human (11). The first case of SARS-CoV-2 infection was reported in Wuhan, China, on 31st December 2019 with the presentation of symptoms of atypical pneumonia. This case was further confirmed to be caused by the novel coronavirus, SARS-CoV-2 (12). According to the WHO, as of 7:54 P.M. CEST, 21 May 2021, 165,158,285 cases of COVID-19 have been reported with associated 3,425,017 deaths worldwide. There were 105,647 confirmed cases of SARS-CoV-2 infections in mainland China, including 4,861 deaths. The biggest potential risk for the spread of COVID-19 around the world was closely associated with travel that caused the disease to spread regionally and globally (13). More importantly, researchers found that SARS and COVID-19 share many similarities in terms of their transmission and pathogenicity. Therefore, COVID-19 firstly transmitted from animal to human at the wet store of the sale of seafood and can transmit from humans to humans (14). At this moment, WHO is investigating if Wuhan was the first place of the first infected human. Coronaviruses, like influenza viruses, spread to various animals in nature. Mammals was infected by α-coronaviruses and β-coronaviruses, and gamma-coronaviruses and delta-coronaviruses tend to infect birds. Moreover, the mammals may also be infected with gamma-coronaviruses and delta-coronaviruses. Although still preliminary, current data suggest that bats are the most likely initial source of the COVID-19 outbreak, which begun in December 2019 in Wuhan, China, apparently spreading from a “wet market” to multiple cities and provinces in China (15).

SARS-CoV-2 is a single-stranded sense RNA virus with a genome size of 27–32 KB (125 nm or 0.125 μm) It has a 5′- cap structure and a 3′–poly-A tail (16). Viruses belonging to the same category have some similar properties. The virus has four main structural proteins including spike (S), membrane (M), envelope (E), and nucleocapsid (N) proteins, which are required to regulate the function and viral structure (17). More importantly, N and S are the most important of these four proteins. N helps the virus to properly form the capsid and the entire viral structure. S helps the virus attach to the host cell (18, 19). The S protein consists of three parts, including a large outer domain, a single channel transmembrane anchor and a short intracellular tail. These three regions can accurately anchor the host cell. In these segments, the outer domain contains two subunits, the S1 receptor binding subunit and the S2 membrane fusion subunit. These subunits locate in the clove-trimer or crown structure, which is why it is named corona virus (corona = crown) (20).

The usual symptoms of COVID-19 include fever (83–98%), cough (59–82%), shortness of breath (19–55%), and muscle ache (11–44%), which are similar to those of SARS and MERS, especially the receptor binding domain (RBD) and the receptor binding motif (RBM) in the viral genome (21, 22). Fever is not the initial symptom in all patients, and some patients experience sore throat, rhinorrhea, headache, and confusion in the days preceding the fever (23). In addition, the characteristics of fever have not been fully defined, with a small proportion of patients presenting with hemoptysis and a large proportion without associated symptoms (24–26). Hemographic aspects, patients have normal or low white blood cell counts, lymphopenia or thrombocytopenia, and elevated C-reactive protein levels (23, 25, 27). Fever and upper respiratory symptoms with leukopenia or lymphopenia should be suspected, especially in patients with a history of travel to epidemic areas or close contact. More importantly, Genomic analysis showed that SARS-CoV-2 is closely related to SARS-CoV, with which it shares about 79% of its genome (28, 29). Angiotensin-converting enzyme 2 (ACE2), as their common receptor, reemerges as a hotspot owing to its indispensable role in facilitating cellular entry of SARS-CoV-2 and SARS-CoV (30), which indicates that a similar pathogenic mechanism is involved in both viral infections. Other similar co-receptors may include Neuropilin 1 (31) and CD4 (32). Interestingly, ACE2 protein is expressed in all organs of the human body, mainly distributed in the main target organs of coronavirus such as lung, kidney and intestine (33, 34). There are clinical reports of male patients experiencing problems such as erectile dysfunction (35) and sexual anhedonia (36) following recovery from infection (low libido). Furthermore, SARS-CoV-2 infects host cells through the ACE2 receptor, resulting in COVID-19-associated pneumonia, as well as causing acute myocardial damage and chronic damage to the cardiovascular system (37).

**Figure 1:**
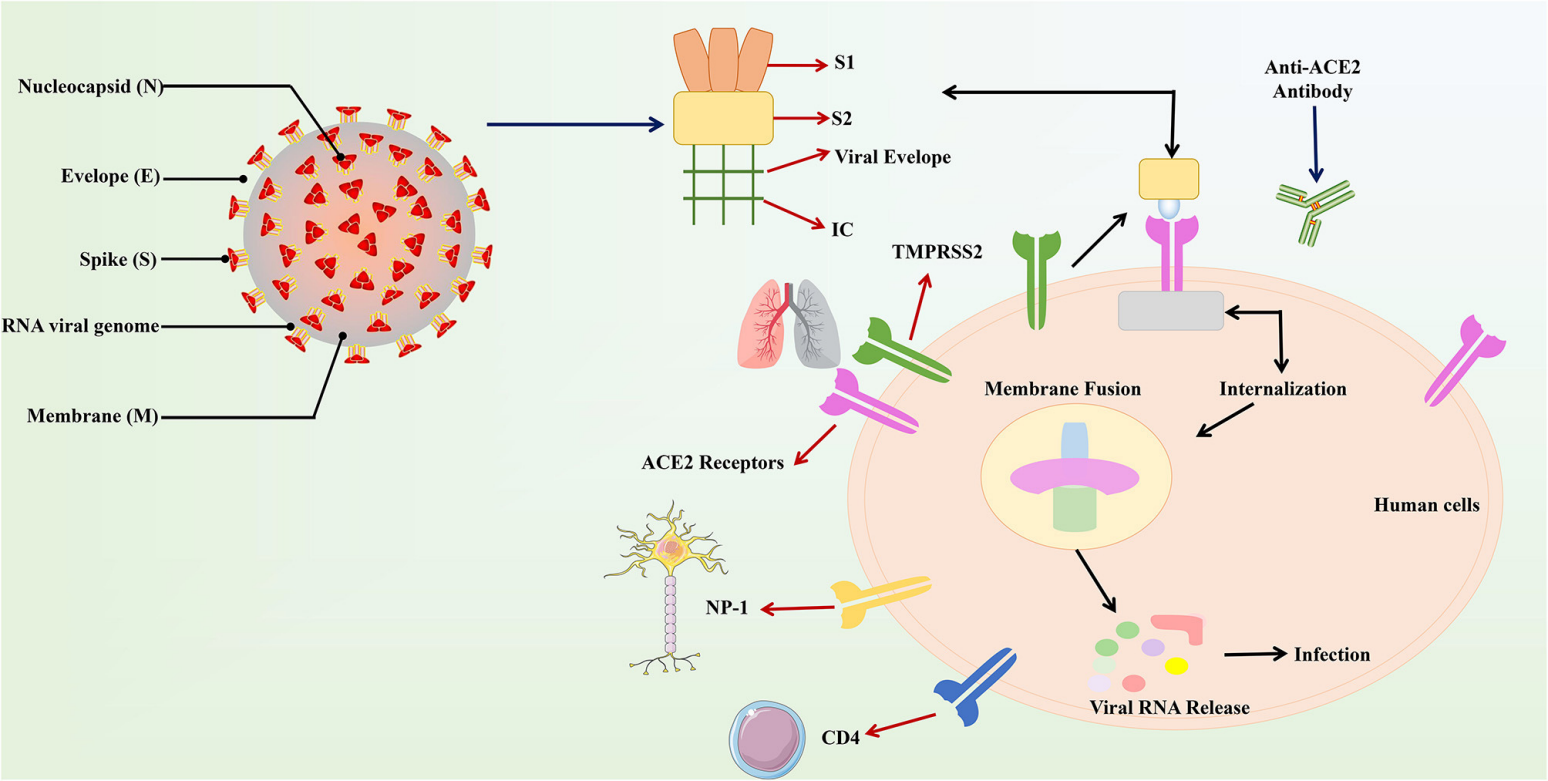
The structure and combination of COVID-19 virus and ACE2. The COVID-19 virus was described in the above figure. In the structure of the virus, the S protein plays a major role in the binding of the virus to the host recipient cell. The S protein is composed of the S1 receptor binding subunit and the S2 membrane fusion subunit. Then S2 subunit is internalized on the ACE2 receptor connected to human host cells and produces the membrane fusion among the viral subunit and the ACE2 receptors. This results in the release of viral RNA into the host cell and causes respiratory infections.

Noteworthy, it has also been suggested that the mechanism of action of COVID-19 in human infections is similar to SARS. According to the reports, an amino acid residue (Gln493) is the main component RBM of the SARS-CoV-2. Moreover, Gln493 promotes the binding of viral S proteins to viruses and fusion into ACE2 proteins in human cells, particularly in the lungs, which can lead to respiratory infections in humans (38). The structure and binding of S protein and ACE2 are described (Figure 1). The easiest and most direct way to fight SARS-CoV-2 is to prevent the virus from entering the cell because the method has been used in previous same kind viruses (19). The key advantage here is that the host ACE2 protein will not change, so there is no need to worry about favorable mutations that may hinder drug development (39).

However, due to the novel nature of the virus, there is no effective drug treatment for COVID-19, so it is particularly important to understand the transmission method of COVID-19, and thereby preventing COVID-19 infection. Considering the severity of the COVID-19 outbreak all over the world, it is urgent to understand transmission pathways to control the spread of the disease to susceptible groups and to identify effective treatments. Hence, in order to control infections and develop effective management systems to deal with virus outbreaks, we should understand the ways of COVID-19 infection and evaluate the similarities and dissimilarities of the SARS-CoV-2 with the viruses that have caused outbreaks in the past. Based on the accumulated data and knowledge on the previous coronavirus infection, this review aims to fill the knowledge gap about the spread ways of COVID-19 and may better help prevent COVID-19 infection.

## Pathogenesis and Epidemiology

### Human Coronaviruses

Coronavirus belong to single-stranded zoonotic RNA virus that initially causes a common cold and further causes respiratory, intestinal, liver, and nervous system symptoms (40, 41), including HCoV-OC43, HCoV-229E, SARS-CoV, HCoV-HKU1, MERS-CoV and HCoVNL63 (42, 43). SARS-CoV-2 is the seventh reported coronavirus in human. Before the SARS-CoV outbreak, these infections were self-limiting in nature. Therefore, HCoV-229E and HCoV-OC43 coronavirus infections have been controlled half a century ago. After the SARS-CoV outbreak, HCoV-NL63 and HCoV-HKU1 were extracted and then controlled (5, 44). Approximately 15–30% of human respiratory infections are caused by these viruses each year. And individuals with weakened immunity are the most vulnerable groups, such as newborns, the elderly, and other chronic comorbidities.

In November 2002, the first known case of SARS occurred in Foshan, China. New cases emerged in mainland China, and by February 2003, more than 300 cases had been reported, around one-third of which were in health care workers (45). In March 2003, the WHO established a network of laboratories to determine the causative agent of SARS. A remarkable global effort led to the identification of SARS coronavirus (SARS-CoV) in early April of that year (46, 47). SARS-CoV is considered to be the most serious disease compared with other coronaviruses. The mortality rate of the SARS-CoV outbreak is 9%. By July 2003 and after a total of 8,096 reported cases, including 774 deaths in 27 countries. More importantly, the mortality rates among the elderly are as high as 50%. SARS not only has a high fatality rate but also causes a severe global economic recession, especially in Southeast Asia and Toronto, Canada (48). After the outbreak of SARS, it gradually spread to more than 24 countries. More importantly, the researchers demonstrated that the SARS-CoV sequence was found in Chinese horseshoe bats, and these bats were infected with a related virus before the outbreak, suggesting that Chinese horseshoe bats are the main reason for the SARS-CoVs outbreak (49, 50). SARS-CoV enters host cells by a mechanism involving the interaction between its S protein and human angiotensin-converting enzyme 2 (ACE2) expressed on host cells in the lung (51). During entry, S is cleaved into an N-terminal S1 region that mediates binding to ACE2 and a fusogenic C-terminal S2 region that mediates viral and host cell membranes (52, 53). The region designated S791 in the study corresponds to amino acids 620–900 of the S protein, which contains the cleavage sites for the S1 and S2 domains (54). S791 also contains an epitope recognized by serum samples from Vietnamese SARS convalescent patients (5). S protein is capable of inducing protective immunity (55–57). However, an antibody targeting the S1 domain enables antibody-dependent enhancement of SARS-CoV entry into host cells when the amino acid sequence of the S protein is altered (58, 59).

In June 2012, 10 years after the first emergence of SARS-CoV, a man in Saudi Arabia died of acute pneumonia and renal failure. A novel coronavirus, MERS-CoV was isolated from his sputum (6). A cluster of cases of the severe respiratory disease had occurred in April 2012 in a hospital in Jordan and was retrospectively diagnosed as MERS (60). According to WHO, since April 2012, 2019 laboratory confirmed cases of MERS-CoVs infection had been reported by January 2020. The lethality rate is 34.3%, with 866 confirmed deaths, the majority occurring in Saudi Arabia (2,121 cases, including 788 deaths, with a lethality rate of 37.1%) (61). Studies found that MERS-CoVs was closely associated with HKU4 and HKU5 (62). Therefore, MERS-CoVs is considered to be derived from bats and is similar to the source of SARS. Serum test results of MERS-CoV antibodies in dromedaries in Middle Eastern countries indicated that the intermediate host of MERS-CoV is camel (63). Importantly, MERS-CoVs were also found on humans and camels in Saudi Arabia and can be transmitted to humans through camels (64, 65). At the end of December 2019, an outbreak of mysterious pneumonia occurred in Wuhan City, Hubei Province, China (66, 67). It was observed that the etiologic agent of the disease was a novel CoV, previously designated 2019-nCoV by WHO and, more recently, SARS-CoV-2 by the International Committee for Taxonomy of Viruses (68). The outbreak appears to have started from a single or multiple zoonotic transmission events at a wet market in Wuhan where game animals and meat were sold (24), and then spread rapidly worldwide. At the latest count on 21st May 2021, the number of confirmed infected cases worldwide has exceeded 165,158,285 in 200 countries with cases reported in all continents and the global reported death toll has risen to 3,425,017. SARS-CoV-2 is a beta-coronavirus like the two other viruses that have caused fatal infections over the last 20 years: SARS-CoVs and MERS-CoVs. More importantly, the transmission from person to person was soon recognized due to the various cases of COVID-19 within families and among people who did not go to the Huanan market (69).

### Epidemiology of COVID-19: Comparison With MERS and SARS

**Figure 2:**
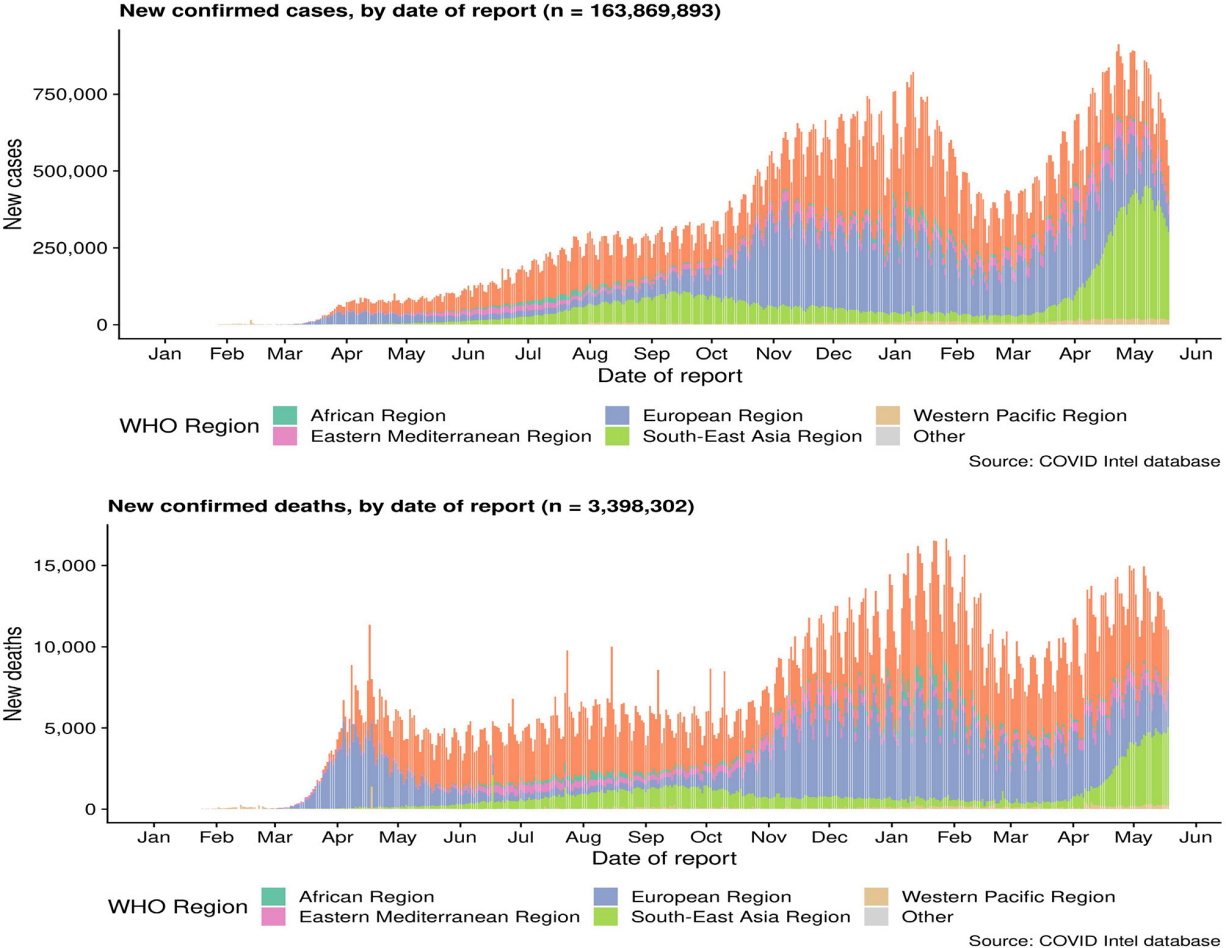
Cumulative confirmed cases and death cases of COVID-19. Data are from the World Health Organization.

As of April 29, 2020, a total of 3,061,615 cases of COVID-19 occurring in at least 170 countries and territories were reported, with ~7% of mortality rate (213,577/3,061,615). A prior overview from China that included 72,314 confirmed, suspected, and asymptomatic patients revealed several important epidemiological features of COVID-19 (70). Most patients were between 35 and 55 years old, and children and infants are less infected (71, 72). A research on the dynamics of early transmission of the virus reported that the median age of patients was 59 years ranging from 15 to 89 years with the majority being males (59%) (13). People with low immune function, especially the elderly, and those with renal and liver dysfunction were vulnerable (13). SARS-CoV2 was found to have higher rates of transmissibility and pandemic risk than SARS-CoV, as the global effective reproductive number (R) of SARS-CoV2 is 2.9, which is much higher than SARS's R (1.77). Another study demonstrated that SARS-CoV2 is between 2.6 and 4.71. The incubation period of SARS-CoV-2 is about 4–8 days after infection (23, 73) (Figure 2).

### Transmission of COVID-19: Comparison With MERS and SARS

Previous reports demonstrated that the interpersonal transmission of SARS-CoV and MERS-CoV was mainly transmitted through hospitals. Forty to one hundred percentage of MERS cases in individual outbreaks were associated with hospitals. In addition, similar observations were performed for some of the SARS clusters (74, 75). Researchers showed that 13–21% of MERS cases and 22–39% of SARS cases were transmitted by family members. Notably, transmission between patients occurred in 60–80% of MERS cases. Moreover, infection of health care workers by SARS-CoV-infected patients was very frequent (33–42%) (74). Therefore, the main reason for hospital spread to become the main mode of transmission is that a large amount of virus shedding only occurs after symptoms appear (76, 77), when most patients are already seeking medical care (78). An analysis of the surface of the hospital after the treatment of MERS-patients revealed that viral RNA is prevalent in the environment within a few days after the patient test is no longer positive (79). In addition, many SARS or MERS patients are infected through super spreaders (74, 77, 80–82).

**Figure 3:**
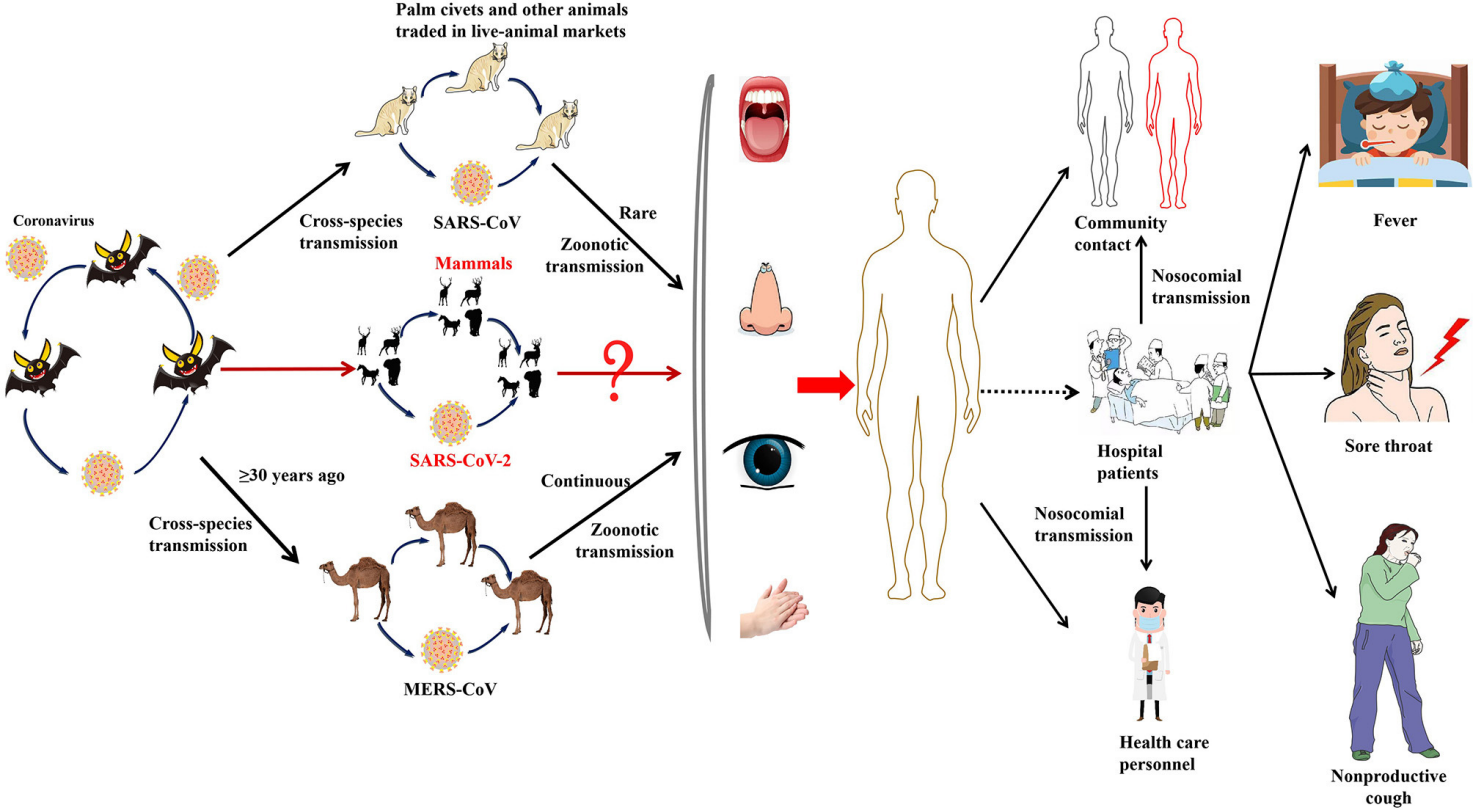
The emergence of SARS-CoV-2, SARS-CoV, and MERS-CoV. Bats harbor a wide range of coronaviruses, including SARS-CoV-like and MERS-CoV-like viruses. SARS-CoV crossed the species barrier into masked palm civets and other animals in live-animal markets in China; genetic analysis suggests that this occurred in late 2002. Several people in close proximity to palm civets became infected with SARS-CoV. A MERS-CoV ancestral virus crossed the species barrier into dromedary camels; serological evidence suggests that this happened more than 30 years ago. Abundant circulation of MERS-CoV in dromedary camels results in frequent zoonotic transmission of this virus. SARS-CoV-2, SARS-CoV, and MERS-CoV spread between humans. However, the main mode of transmission of COVID-19 is unclear.

On the other hand, it is speculated that the wet animal market in Wuhan, where live animals are sold, may be the origin of SARS-CoV2 zoonotic. Therefore, scientists are working hard to find a host reservoir or an intermediate vector in which the infection could spread to humans (11). Previous studies showed that the possible SARS-CoV2 reservoirs were the two snakes. However, the researchers only clearly demonstrated that mammals and birds were the reservoirs of coronavirus to date. SARS-CoV2, the causative agent of this respiratory disease, was identified and its genome is fully sequenced. The genomic sequence of SARS-CoV-2 showed a similar, but distinct genome composition of SARS-CoV and MERS-CoV, indicating that bats are the most likely connection between COVID-19 and humans (83). Several studies have shown that transmission from person to person is a possible way for COVID-19 infection to spread. Furthermore, SARS-CoV2 has infected many people who have not visited the wet animal market in Wuhan, further proving that COVID-19 can be transmitted from person to person (84). Three routes of transmission exist for SARS-CoV-2 in the human population: (1) inhalation of droplets generated by an infected person (2) close contact with an infected person (3) contact with an object surface contaminated with SARS-CoV-2 (85). SARS-CoV2 can be released when coughing, talking and sneezing of infected person. Droplets containing the virus can infect others if they do not follow a safe distance (86). Figure 2 shows the current global infection situation, indicating that the transmission rate of SARS-CoV2 is extremely fast. More importantly, recent studies reported that the newborn from infected mothers were not infected with SARS-CoV-2, suggesting that SARS-CoV-2 could not be transmitted vertically from mother to newborn. Nonetheless, the pregnant mother had a cesarean section, so it was still uncertain whether it would spread during vaginal delivery. This study is extremely important because pregnant women are more susceptible to respiratory infections and extreme pneumonia (83). In addition, previous studies have shown that weather has an important impact on infectious disease transmission, including but not limited to influenza and SARS. For example, sudden changes in ambient temperature increased the risk of SARS transmission (87, 88). Therefore, it is hypothesized that COVID-19 transmission may decrease or even disappear when the temperature and UV radiation increase in the summer. However, Ye et al. demonstrated that the spread ability of COVID-19 would not change with increasing temperature and increasing UV exposure. In addition, they did not find significant associations of relative humidity, maximum temperature and minimum temperature with cumulative incidence rate or R0 of SARS-CoV2, suggesting that it might be premature to count on warmer weather to control COVID-19 (89). SARS-CoV2 binds to the receptor expressed by the host cell is the first step of infection, and then fuse with the cell membrane. The primary target of SARS-CoV2 was the lung epithelial cells. Studies have shown that the combination between ACE2 and the receptor binding domain of the viral peak is the main link for SARS-CoV-2 to achieve human-to-human transmission (83). More importantly, the sequence of receptor binding domain of SARS-CoV2 is similar to SARS-CoV, indicating that the ACE2 receptor is the main factor for SARS-CoV2 to enter the host cell (90) (Figure 3). In addition, recent studies have found that the receptors of SARS-CoV2 may also include neuropilin-1 (NP-1), transmembrane protein serines2 (TMPRSS2), CD4, etc. The existence of these receptors is of great significance to the pathological progress and clinical manifestations of COVID-19. For example, the activation of virus S protein by host cell protease TMPRSS2 is a prerequisite for the invasion of SARS-CoV2 (91). For another example, the sharp decrease of CD4 + T cell count in patients with COVID-19 is associated with poor clinical outcomes (92). Moreover, NP-1 may be a way for SARS-CoV2 to enter the nervous system, which is related to the olfactory loss symptoms in some patients (32).

### Clinical Presentation of SARS-CoV-2: Comparison With SARS-CoV and MERS-CoV

The data obtained from different populations in the world and 31 provinces in China indicated that there are many similarities between the clinical symptoms of SARS-CoV-2 and the clinical symptoms of SARS-CoV infection (93, 94). The median age of the infected patients was 47 years. Therefore, males account for 58.2% of infected patients, and the mean incubation time was 3.0 days (range: 0–24.0 days) (95). More importantly, dyspnea develops within a median of 8 days after the onset of illness (range of 5–13 days), while in others, respiratory distress may be absent in some infected patients (73). The most frequently reported symptoms include cough-producing phlegm, runny nose, myalgia, fever, pneumonia, complicated dyspnea, body ache, fatigue, nausea, vomiting, diarrhea, conjunctivitis, skin lesions and anosmia, while the less widely documented symptoms include headache, vomiting and hemoptysis, which were similar to that of SARS-CoV infection (96, 97). Lymphocytes and white blood cells decreased, fever and new pulmonary infiltrates on chest radiography with no improvement in symptoms after 3 days of antibiotic treatment were considered to be the main clinical features that defined COVID-19 (8). Importantly, the intensive care may be required by 3–29% of infected patients. Severe patients may rapidly develop multiple organ dysfunction and eventually lead to death, and infected patients with shortness of breath and hypoxemia may rapidly develop acute respiratory distress syndrome (ARDS), severe sepsis accompanied by shock and even multiple organ dysfunction syndromes (MODS) within a week (73, 93).

Recently chest computed tomography (CT) of case series (*n* = 18) of infected patients were documented. They reported that typical CT findings included bilateral pulmonary parenchymal ground-glass and consolidative pulmonary opacities, sometimes with a rounded morphology and a peripheral lung distribution (98). Notably, lung cavitation, discrete pulmonary nodules, pleural effusions, and lymphadenopathy were absent (98). In addition, researchers found that fever, followed by fatigue, dry cough, myalgia, and dyspnea are the most common symptoms of infected patients in case series (n = 138) of COVID-19 in a hospital in Wuhan, China. Most infected people are almost 50 years old. The majority of infected patients who were admitted to ICU are the elderly and others with comorbidities, and the fatality rate reached 4.3% among patients in the study (73). Besides, the results of 41 laboratory-confirmed cases of COVID-19 in Wuhan, China showed that males, combined with other comorbidities, and exposed to Huanan seafood market were the main infected person. The symptoms were similar to the previously reported cases (28). It is worth noting that the direct damage of the virus to the intestine leads to the gastrointestinal symptoms of COVID-19, rather than the immunopathogenic response to host lung infection (99). Studies showed that the virus can be shed in the gastrointestinal tract and fecal-oral transmission due to the ACE2 that was expressed in the human gastrointestinal epithelial cells (100). Collectively, the epidemiology, transmission and clinical presentations of the most important coronavirus outbreaks were described in Table 1 (SARS-CoV-2, SARS-CoV, and MERS-CoV).

**Table 1:** Characteristics of patients with SARS-CoV-2, SARS-CoV, and MERS-CoV.

	**SARS-CoV**	**MERS-CoV**	**SARS-CoV-2**
Disease	SARS	MERS	COVID-19
Outbreak beginning date	November 2002	April 2012	December 2019
Location of the first case	Guangdong, China	Saudi Arabia	Wuhan, China
Confirmed cases	2,519 (From 2012 until January 31, 2020)	8,096	4,179,479 (13 May 2020)
Fatality rate	~10%	~36%	6.8 %
Time to infect first 1,000 people (Days)	130	903	48
Latency (Days)	2–7	5–6	7–14
Incidental host	Masked palm civets	Dromedary camels	Malayan pangolin
Contagious period	10 days after onset of disease	When virus could be isolated from infected patients	Unknown
Transmission	Respiratory droplets; Close contact with diseased patients; Fecal-oral; Aerosol.	Respiratory droplets, Close contact with diseased patients/camels Ingestion of camel milk	Touching or eating an infected, yet unidentified animal. Human-to-human transmission occurs through close contact
Reservoir	Bats	Bats	Bats
Radiologic features	Diverse from focal faint patchy ground-glass opacities to bilateral ill-defined air space consolidations on plain chest radiograph. Non-specific to distinguish between three different diseases		
**Clinical presentation**			
Age, years (range)	39.9 (1–91)	53 (36–66)	47.0 (all spectrum of age)
Male: female ratio	1:1.32	2.07:1	1.41:1
Fever	99–100%	73 ± 4%	85.8% (%)
Nausea or vomiting	19–20%	–	4.6%
Nausea and/or vomiting	19–20%	–	5.0%
Fatigue	31%	–	28.6%
Myalgia	49–61%	–	15%
Expectoration	NR	–	13%
Headache	35.4–55.8%	–	8.4%
Non-productive cough	24.9–74.8%	80.4 ± 5%	68%
Dyspnea	39.6–42.3%	–	46%
Sore throat	13%	38.7 ± 11%	14%
Headache	35.4–56%	–	7.5%
Diarrhea	19.8–25.1%	9.5–19.8%	6.0%
Dizziness	4–43%	–	4.0%

*The average of some clinical presentations for MERS-CoV was not available in the literature*.

## Prevention and Control of COVID-19

The methods and strategies for prevention and control of COVID-19 are reported at three levels, including national level, case-related population level, and general population level. At the national level, the National Health Commission of China issued “Announcement No. 1,” which formally incorporated COVID-19 into the management of Class B legal infectious diseases and allowed the implementation of prevention and control measures for Class A infectious diseases (101, 102). In order to prevent and control the spread of COVID-19, observation programs and isolation treatment can be carried out according to this policy in medical institutions (103). On January 22, 2020, the National Health Commission of China issued the “Guidelines for the Prevention and Control of New National Coronavirus Infection (COVID-19),” and requires medical institutions to do a good job in the prevention of nosocomial infections (101). On January 28, 2020, the “Guidelines for Rapid Prevention and Control Measures” was issued by the National Health Commission to effectively curb the spread of disease through the “Great Isolation and Disinfection” program during the Chinese New Year (101). Importantly, in order to better suppress the spread of COVID-19, the National Health Commission issued a series of targeted measures for the elderly population (released on January 31, 2020) and rural areas (released on January 28, 2020) (101). At present, the case isolation, identification and monitoring of contacts, environmental disinfection and the use of personal protective equipment were performed to prevent the spread of SARS-CoV-2 in 2019 in China (104). So far, infected patients treated with antiviral therapy have not been confirmed to be infected with COVID-19 again. Moreover, experts recommended that appropriate symptomatic treatment and supportive care should be taken for the treatment of patients with COVID-19. Additionally, studies reported that the psychological health issues and nosocomial infection were linked to COVID-19. It is recommended to take measures to minimize nosocomial infection including prevention and control awareness training, isolation, disinfection, graded protection in infected areas, and case report protection (105). More importantly, in terms of mental health, psychological intervention is recommended for medical personnel, suspected cases and confirmed cases (106).

At present, a total of 7 kinds of COVID-19 vaccines have been certified by the WHO. At present, the number of people vaccinated with the COVID-19 vaccine has exceeded the number of infected people, but it is still far from establishing an effective immune barrier. Therefore, avoiding exposure to SARS-CoV2 is the best preventive measure for the general population. The airborne measures and other protections were discussed and suggested for protection. According to CDC recommendations, the main strategies for preventing the spread of COVID-19, include(1) Covering sneeze or cough with a tissue and discarding the contaminated tissue in a contained trash; (2) Maintain social distance of six feet and above; (3) Avoiding travel to high risk areas; (4) Wash hands with soap and water for at least 20 s; (5) If soap was unavailable, use a disinfectant that contains at least 60% alcohol; (6) Avoiding hand contact with facial components; (7) Cleaning and disinfecting repeatedly touched surfaces (107). Moreover, the detailed recommendations of the use of face masks were issued by WHO within the population (108). Training courses on the correct use of surgical masks or N95 masks and various personal protective equipment were held for the staff. Those who failed to pass the mask use training twice received the training of powered air purification respirator (PAPR). This allows everyone in the team to be ready to respond to cases that may arise (109). Importantly, it is recommended that particulate masks should be used when medical personnels perform aerosol generation procedures, such as licensed N95 or FFP2 masks. The surgical masks should be used when medical personnels provide treatment for suspected or confirmed patients. It is necessary for all individuals, with or without symptoms of respiratory infection, to wear masks in public as there may be a significant number of people with asymptomatic infection in the population (110). In addition to papers published in scientific journals, the Chinese Center for Disease Control and Prevention also provides guidelines to increase public awareness of COVID-19 prevention and control, including causes of illness, how to choose and wear masks, correct hand washing methods, preventive measures taken in different places (such as homes, public transportation and public spaces), disinfection methods and home medical monitoring (110).

## Ongoing Clinical Trials

**Figure 4:**
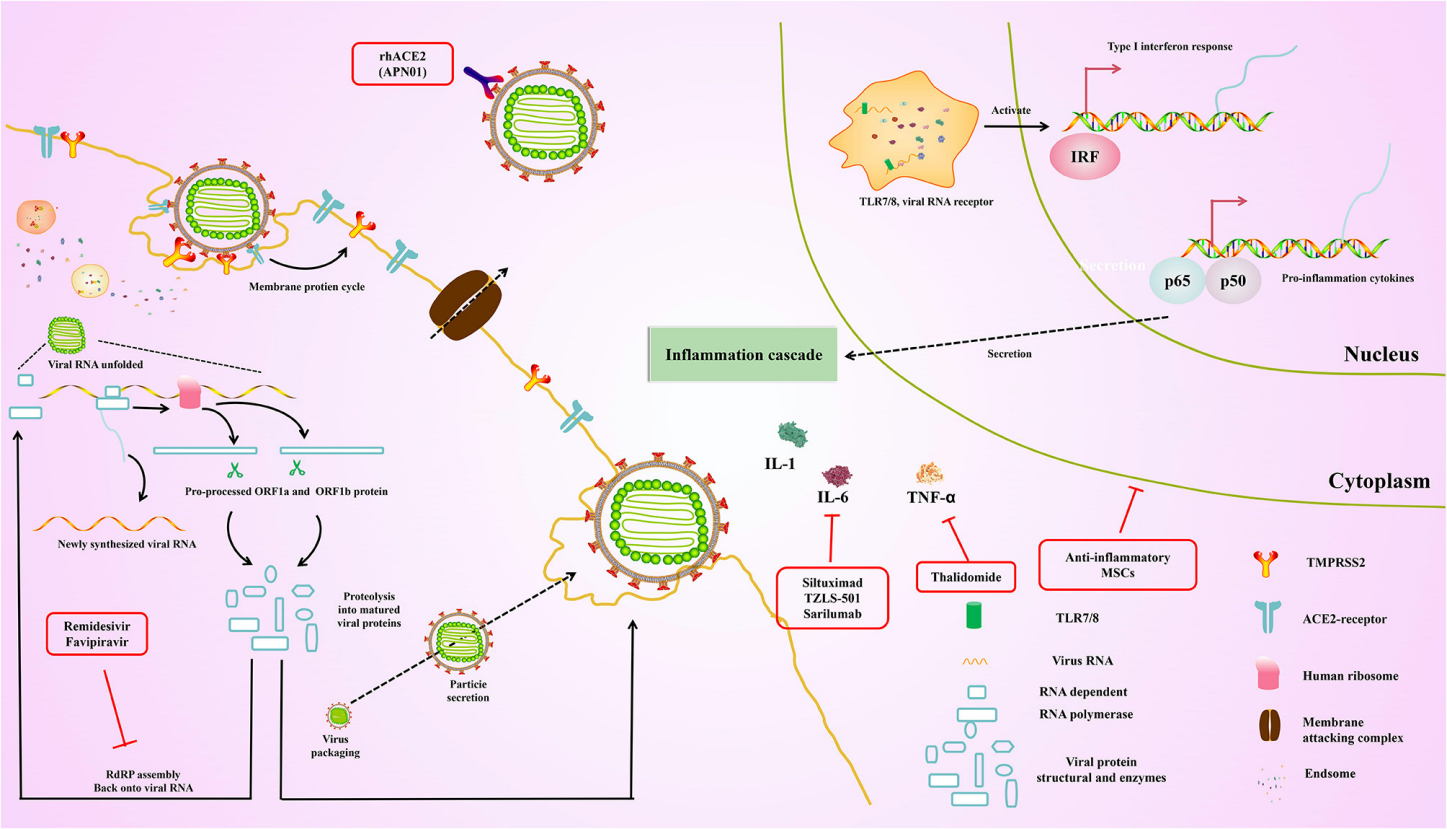
Overview of the repurposed therapeutic drugs undergoing clinical trial against COVID-19 in the context of host pathways and virus replication mechanisms.

Currently, existing antiviral drugs are shown to be ineffective in treating COVID-19 pneumonia. However, several clinical trials of potential antiviral drugs are initiated. The therapies can be divided into two categories according to their target. The first category is acting on coronavirus directly, either through suppressing viral enzymes, which regulate genome replication or *via* preventing the virus from entering the host cells. Another aims to regulate the human immune system, either *via* elevating the innate immune responses or through blocking the inflammatory processes which cause liver injury. These drugs were originally designed to inhibit other pathogens and are rapidly being used in current COVID-19 trials. At the same time, the specificity of specific vaccines and antibodies against SARS-CoV-2 was tested in several trials. Here, we summarize ongoing treatment options that may lead us to combat COVID-19 (Figure 4).

### Inhibiting the RNA-Dependent RNA Polymerase

#### Remdesivir

The specific drug, which belong to the adenosine nucleotide analog, is a promising and potential anti-RNA virus broad-spectrum antiviral drug (111). Therefore, it can enter into the nascent viral RNA and further inhibit RNA-dependent RNA polymerase, leading to the premature termination of the viral RNA, thereby inhibiting the replication of the viral genome. Remdesivir was originally used against the Ebola virus which was developed by Gilead Sciences (USA), and has gone through clinical trial during the recent Ebola outbreak in the Democratic Republic of Congo (112). Although there was no significant effect against Ebola in this trial, the drug has been shown to be safe in humans, which was introduced into clinical trials immediately against COVID-19 in an emergency (112). Previous studies have shown that remdesivir could be used in treatments against SARS-CoV and MERS-CoV (113, 114). Importantly, recent studies demonstrated that the SARS-CoV-2 was also inhibited (115). According to the reports of the first COVID-19 patient in the United States, it was proved that remdesivir significantly improved the condition of patients on the 8th day without s any noticeable adverse reactions (116). Another recent study has shown that remidesivir scored 0.77 μM at half-maximal concentration against COVID-19 and blocked viral infection (73, 115, 117). On October 22, 2020, remdesivir was approved by the U.S. FDA as the first COVID-19 drug across the United States.

#### Favipiravir

Favipiravir is similar to remidesivir which was developed by Toyama Chemical (Fujifilm Division, Japan). Of note, favipiravir is structurally similar to endogenous guanine, thereby resulting in the inhibition of RNA-dependent RNA polymerase. In addition, the competitive inhibition greatly reduces the effectiveness of virus replication (118). Although it is used against influenza, there are still fewer clinical studies supporting favipiravir in treatment for COVID-19 compared with the remdesivir. Nevertheless, favipiravir and interferon-α are given simultaneously to increase immunity and suppress the virus. Hence, the synergistic effect of favipiravir and interferon-α (ChiCTR2000029600) was assessed *via* recruiting the patients. Indeed, favipiravir was approved by China's National Medical Products Administration in March 2020 as China's first anti-SARS-CoV-2 drug due to its proven efficacy and low side effects, which was demonstrated by the clinical trial.

### Blocking Virus–Cell Membrane Fusion

#### Recombinant Human ACE2 (APN01)

The entry of SARS-CoV-2 is inhibited by the soluble recombinant human ACE2 (rhACE2) *via* preventing the interaction between S protein and cellular ACE2. Currently, the recent studies reported that the RNA replication of SARS-CoV-2 is blocked by rhACE2 in cellular and embryonic stem cell-derived organoids, and its therapeutic effect by up to 1,000–5,000 times (119). More importantly, the rhACE2 keeps the substrate away from the associated enzyme, leading to the downregulation of serum level of angiotensin II, further inhibiting the ACE2 receptor activation, which could maintain the pulmonary vascular integrity and inhibit ARDS (120). Recently, APN01 was developed by Apeiron Biologics, and the phase II trial for ARDS is being performed. Importantly, the biological and physiological function of rhACE2 in SARS-CoV-2 is now assessed in China (NCT04287686). especially in the treatment of ARDS. In addition, the safety and tolerability of APN01 were also evaluated by Apeiron Biologics. Interestingly, the biological and physiological function of rhACE2 in SARS-CoV-2 is assessed *via* detecting the plasma level of angiotensin II and angiotensin 1–7 of bioproducts.

#### Arbidol Hydrochloride (Umifenovir)

Arbidol has been approved in Russia and China as an entry inhibitor against influenza viruses and arboviruses (121). Arbidol inhibits the fusion of viral membrane and endosome after endocytosis by targeting hemagglutinin (HA), which is the main glycoprotein on the surface of influenza virus. Currently, the clinical trial of a signal agent is underway in China (NCT04260594, NCT04255017). Additionally, a randomized clinical trial has shown that arbidol has more evidently therapeutic effects compared with favipiravir (ChiCTR2000030254) (122).

### Enhancing the Innate Immune System

#### Natural Killer Cells

Accumulating reports revealed that the majority were among patients of ≥ 60 years of age, with the ≥ 80 age group characterized by the highest fatality rate (20.3%) among all age groups, which may because of the weakening of the immune system with age. Therefore, enhancing innate antiviral immune response may be an effective antiviral method. Natural killer cells (NK) are an important part of the innate immune system, which promote the sensitivity of immune system to virus. Previous studies have demonstrated that the SARS-CoV2 clearance can be regulated by the pulmonary migration of NK cells and macrophages (123). Additionally, the secretion of cytokines and chemokines are elevated by the innate immune system without the CD8^+^ T cells and antibodies, thus controlling the infection of COVID-19. The phase I trial is being performed to assess whether viral clearance from COVID-19 pneumonia is facilitated by NK cell, which is expected to be completed by the end of 2020. Currently, the related anti-cancer drugs based on NK cells may be used to treat COVID-19 by some companies.

#### Recombinant Interferon

The cells infected by virus secretion type I interferons, which have broad-spectrum antiviral effects against HCV, respiratory syncytial virus, SARS-CoV and MERs-CoV when used alone or in combination with other drugs (124, 125). Currently, clinical trials are performed to evaluate the safety and efficacy of type I interferons against SARS-CoV-2 (NCT04293887).

#### Convalescent Serum

There have been clinical studies showen that in COVID-19 patients convalescent plasma can play a great anti-inflammatory role. The mechanisms include the presence of IgM/IgG neutralizing antibodies that can inhibit viral entry into host cells and activate the host's immune system through antibody dependent cellular immunity. But the choice of the timing of rehabilitation therapy is important. If given in an inappropriate amount of time, cytokine storm syndrome may instead be triggered. When using rehabilitative plasma, the following points should be noted:

1. Immunoglobulins are not completely homologous between individuals.
2. Patients may have more viral load than the immunoglobulins transferred to them, that is, patients need more than one dose to be injected.
3. Transferred Ig may reduce the Ig produced naturally by the patient's immune system.
4. Antibodies produced by the patients themselves and transferred antibodies may form immune complexes, causing infusion reactions that can lead to nephritis or other serious consequences.
5. There is a latent phase for the virus.
6. There may be differences in the subtypes of immunoglobulins between donors and recipients.
7. There is insufficient information on the predominant IgA in the recipient mucosal tissues.

#### Monoclonal Antibodies

Monoclonal antibodies are currently used primarily in the treatment of COVID-19 patients, and the first relevant human study was with LY-CoV555 directed against the spike protein, performed by the pharmaceutical company “Lilly,” which is currently in phase II trials. Recently, monoclonal antibodies such as Bamlanivimab and Casirivimab-Imdevimab were licensed for the treatment of outpatients with mild to moderate infection with SARS-CoV2. Of particular note, the early use of monoclonal antibodies (against IL-6, IL-1β, IL-17, interferon-γ TNF-α) is beneficial in controlling the covid-19 inflammatory storm, attenuating the progression of the condition, and reducing mortality. In addition, monoclonal antibodies are also being tried as vaccines and rapid diagnostic reagents with a bright future (126).

#### Corticosteroids

Studies of corticoid therapy for viral pneumonia and acute respiratory distress syndrome have had mixed results. However, a randomized evaluation of COVID-19 treatment (recovery) trial, which randomized 2,104 patients with COVID-19 to receive 6 mg dexamethasone daily for up to 10 days and 4,321 patients receiving usual care, found that dexamethasone reduced 28-day-all-cause mortality [21.6 vs. 24.6%; Age adjusted rate ratio, 0.83 (95% CI, 0.74–0.92); *P* < 0.001] By contrast, in patients with a shorter duration of symptoms and no supplemental oxygen requirement, there was no benefit (and potential for harm). A retrospective cohort study of 201 patients diagnosed with COVID-19 and acute respiratory distress syndrome in Wuhan, China, reported that treatment with methylprednisolone was associated with a reduced risk of death [hazard ratio, 0.38 (95% CI, 0.20–0.72)]. Overall, corticoid administration is beneficial for the control of inflammatory storm and is suitable for the treatment of severe COVID-19 patients (127).

## Future Perspectives

Some strict measures need to be implemented to reduce the spread of COVID-19 from person to person, especially vulnerable people, including infants, health care providers and the elderly. In addition, the Chinese Center for Disease Control and Prevention issued protective guidelines for medical personnel, health care professionals, public health individuals and researchers associated with COVID-19 (106). Among the cases, the majority were the elderly and characterized by the highest fatality rate due to the poor immune system, which causes the virus infection to progress faster (128). The public facilities should be disinfected regularly. In addition, contact should be avoided when handling infected samples, in order to prevent infection COVID-19 (129). The prevention and control measures were implemented in the world, especially the United States, China, India, and the UAE, such as blockades and travel screening in order to control the spread of COVID-19. In addition, we should closely monitor the epidemiological changes of COVID-19 infection, such as possible transmission routes, subclinical infections, adaptation, evolution, possible intermediate animals and reservoir and transmission between humans. There are still many problems to be solved. Including the identity, the number of people tested, how many of the test people are positive, whether this ratio remains the same or changes. So far, only a few pediatric cases have been reported due to lack of sensitivity or insufficient testing.

## Conclusion

SARS originated from a wildlife trade in Shunde, Guangdong, China, where the virus could be transmitted by contact with patient droplets or respiratory secretions. After the virus enters the human body, it binds to the ACE2 receptor of lung epithelial cells, and utilizes the host cell system for replication, transcription, and translation. The way MERS-CoV infects cells is extremely similar to that of SARS-CoV, but unlike the latter, the receptor that MERS-CoV binds to in humans is DPP4. The intermediate host for MERS is the dromedary camel, where the virus is transmitted from bats to dromedaries and then from dromedaries to humans. Like SARS-CoV, the receptor for SARS-CoV-2 also includes ACE2. SARS-CoV-2 can bind to ACE2 receptors of animals such as humans, bats, and pigs, suggesting that these animals may be intermediate hosts of SARS-CoV-2. These virus outbreaks will permanently exist or even undergo secondary outbreaks due to the genetic changes that are inevitable in the process of virus evolution. Therefore, developing effective antiviral therapeutics and vaccines for COVID-19 is urgent. To achieve this goal, we need to fully understand the molecular mechanism and pathogenic principle of the virus. Analysis of the correlation and differences between the SARS-CoV-2 epidemic and previous coronavirus outbreaks, particularly the integrated use of biochemical, proteomic, genetic, structural, bioinformatic, virological, and imaging methods to find pan coronavirus therapeutic targets, may provide us with clues for the development of new COVID-19 therapeutics and vaccines. Several pan coronavirus therapeutic targets such as Tom70, sigma-1 (130), etc. have been reported. As a long-standing effort has been devoted to the development of a universal influenza vaccine in general, the development of a universal coronavirus vaccine should also be put on the agenda, which is perhaps less difficult than that of a universal influenza vaccine.

## Author Contributions

JY, HZ, and HF drafted the manuscript. LZ and LW revised the manuscript. BC, XZ, and CZ reviewed and modified the manuscript. SC reviewed and modified the Figure. All authors agreed on the final version.

## Funding

This study was supported by the National Natural Science Foundation of China (Grant Nos. 81673444, 81473223, and U1803129).

## Conflict of Interest

The authors declare that the research was conducted in the absence of any commercial or financial relationships that could be construed as a potential conflict of interest.

## Publisher's Note

All claims expressed in this article are solely those of the authors and do not necessarily represent those of their affiliated organizations, or those of the publisher, the editors and the reviewers. Any product that may be evaluated in this article, or claim that may be made by its manufacturer, is not guaranteed or endorsed by the publisher.
